# Alcohol in Health and Disease

**Published:** 1895-08-17

**Authors:** Tom R. Taylor


### MEDICAL PROGRESS AND HOSPITAL CLINICS.

[*The Editor will be glad to receive offers of co-operation and contributions from members of the profession. All letters should be addressed to* THE EDITOR, THE LODGE, PORCHESTER SQUARE, LONDON, W.]

### ALCOHOL IN HEALTH AND DISEASE.

By TOM R. TAYLOR, M.D., B.S., B.Sc.Lond., F.R.C.S.Eng.

Alcoholic beverages have for centuries formed a topic for controversy and a butt for the invective of moralist and philanthropist. It is not my purpose to pose as either one of these, but rather to consider the use of alcohol apart from its abuse and attendant miseries.

Let us, in the first place, consider the direct effects of alcohol upon the body.

In the mouth, undoubtedly, pleasant drinks of this description do give a relish to food, stimulate the flow of saliva, and so indirectly tend to increase the flow of gastric juice. In the stomach, moderate amounts of these liquids cause increased secretion, and make the necessary movements of the viscus more energetic—in a word, assist digestion. In too great quantity, or in too concentrated a form, it rather tends to inflame the mucous membrane, and greatly interferes with secretion, the most important of all functions, hence the dyspepsia, loss of appetite, and consequent emaciation of many spirit-drinkers.

With moderate doses, the heart beats more forcibly, the blood-pressure rises, the smaller vessels are dilated, and the skin flushes, causing a pleasurable sensation of warmth. Similarly there is a stimulant effect upon the nervous system; association of ideas is more rapid, the conversation becomes more brilliant, and there is a greater tendency to think less of the overhanging cloud than of the proverbial silver lining.

But let the dose be too large, and the erstwhile stimulant becomes a powerful depressant; and death from alcoholic poisoning through shock is not unknown.

A celebrated surgeon tells the story that once, prevailed upon against his better judgment, he consented to operate upon a patient, who refused to take an ordinary anæsthetic, whilst the man was under the influence of large doses of brandy. The difficulties of the operation were tremendous on account of the patient's condition; but so powerful had been the depressant effect upon the nervous system that little if any pain was experienced. This numbing effect of alcohol is, to my mind, the secret of its success in cases of neuralgia, where a few glasses of wine so often relieve the pain. I say a *few* glasses advisedly, for all admit that the dose must be a strong one in order for it to have any effect. The relief is generally ascribed to the stimulation of the nervous system—but *experto crede*. Any good arising is generally speedy, and the sufferer may fall asleep and awake free from any trouble, having at last obtained the rest he needed.

With regard to the excretion of alcohol, there is much contradictory evidence. Certain observers declare that no alcohol is actually made use of and consumed by the body, and that even with the smallest dose it may be detected in the breath and urine. Bouchardat, Sandras, and others state that it is freely consumed, and but little escapes in the urine unless large quantities are taken. Some, at any rate, is consumed and oxidised, and presumably made use of by the body.

Much ink has been spilt upon the question of alcohol as a food. There is no doubt that in sufficiently small quantities it is a true food. But even were it not so, if it serves a useful purpose in the economy, why condemn it? Because of the evils arising from its abuse, should we absolutely banish it? Total abstainers take tea, and often in excess. Yet tea is not a food, and tea in more than moderation is most harmful.

"Tea poisoning" is not very uncommon, with its tremulousness, nervousness, insomnia, constipation, and dyspepsia. But the tea-drinker is respectable, and may from his high pinnacle pity that abandoned creature, the moderate drinker, hurrying along the broad and easy path.

We are now considering alcohol as to its action upon the so-called healthy body. But are we justified in regarding anyone who lives and works in the whirl of excitement, worry, and overwork that passes by the name of life in this the nineteenth century healthy? All more or less suffer from overstrain and anxiety, and it is the latter that kills. As the world grows older the physical part of our nature appears to become more and more subservient and dependent upon our state of mind. See the anxious business or professional man, who complains of "not feeling the thing."

What is the matter? No actual disease can be made out, nor can a name be given to his complaint. He is overtaxed and exhausted, and the overstrung nervous system, incapable of prolonged purposeful action, too readily responds to any external stimulus. In a word, he is in a neurotic state, only too ready to become hypochondriacal or actually to succumb to a definite malady. We moderns burn the candle at both ends, and wonder at the rapidity of combustion. And still we have the supporters of teetotalism putting forward the longevity and healthfulness of the ancient patriarchs and the nomadic shepherd races as an argument in their favour.

Had we been born in a tent and educated in the desert, where quarter-days bring no qualms and where the single-course dinner of dates *à la sauce Spartane* leads to no tradesmen's bills, then, indeed, we might listen with more than respect.

But it is not so. The times have changed, and we with them, and a Bedouin Arab's life would attract but few. One might say that the majority of people now lead a more or less sedentary life—in point of fact, an artificial and unnatural one. The various organs lack the natural stimulus to action which they would have were a more natural existence possible. We find this especially so, perhaps, in the case of the stomach.

Desire for food fails, and some artificial means must be found to stimulate the nerves of taste and the mucous membrane of the stomach. A "tonic" may be prescribed, and this will contain some bitter in the form of a tincture in all probability, *i.e.*, alcoholic and an aromatic substance, and is generally extremely nasty. Now, in sound ale or beer we find these constituents in a much more palatable form. For the stomachs unable to take beer there are the light wines with their slight acidity and well-diluted alcohol, which in a similar manner stimulate the salivary glands, and so, indirectly, the stomach. In cases where the general tone is lowered there is, to my mind, no better tonic than a glass or two of sound burgundy or claret, taken with food. To take alcohol between meals or constantly throughout the day is a mistake, and worse—an insult to the system, which it will sooner or later violently resent. The first pleasurable stimulus quickly passes into one of depression, which leads to a new craving for a something, and that something another nip.

Taken with food, the *bien-être* of a well-digested meal will take the place of this craving. So far it has been as a beverage that we have considered alcohol, but it is also a drug, and some would have us restrict it to this *rôle*. It is in cases of high fever, grave depression, and wasting that we see the *summum bonum* of alcohol.

All agree that it has the power of saving tissue waste and diminishing the oxidative processes. It is most remarkable to observe the large quantities that may be administered in some maladies. In erysipelatous and septic cellulitic conditions it may be exhibited as a rule with the greatest possible advantage. With regard to its exhibition in ordinary febrile conditions, it is not to be given in a routine manner, and if given, the effects upon the patient must be carefully watched. The results will guide the practitioner as to their further administration. Dr. Armstrong's rules are excellent. (1) If the tongue becomes dry, discontinue; if moister, the drug is doing good. (2) If the pulse becomes quicker harm is being done, and the contrary if slower. (3) If the skin becomes moister the antipyretic effect of alcohol is obtained and again good is being done. (4) If the breathing becomes easier continue the drug. With regard then to its use in therapeutics, like all other drugs, the judgment of the prescriber must be used for each and every case. And let me add to the wise physician empiricism and rule of thumb are anathema. Alcohol, though a drug, must be regarded after its centuries of use and abuse, and its almost universal consumption, as a beverage, but one to be made use of with caution. It is to be taken with food, and in its best forms as wholesome wines or ales, not haphazard all day long, and only then is it necessary to those worn out by work or otherwise debilitated. To these latter alone are the words of St. Paul applicable, "Drink no longer water, but use a little wine for thy stomach's sake and thine after infirmities."

